# Impact of virtual reality exercises on anxiety and depression in hemodialysis

**DOI:** 10.1038/s41598-023-39709-y

**Published:** 2023-08-01

**Authors:** Agnieszka Turoń-Skrzypińska, Natalia Tomska, Hanna Mosiejczuk, Aleksandra Rył, Aleksandra Szylińska, Małgorzata Marchelek-Myśliwiec, Kazimierz Ciechanowski, Radosław Nagay, Iwona Rotter

**Affiliations:** 1 Department of Medical Rehabilitation and Clinical Physiotherapy, Pomeranian Medical University in Szczecin, Szczecin, Poland; 2 Clinical Department of Nephrology, Transplantology, and Internal Medicine, Pomeranian Medical University in Szczecin, Szczecin, Poland; 3 Department of Visual Communication, Faculty of Design, Academy of Art in Szczecin, Szczecin, Poland

**Keywords:** Physiology, Health care, Medical research, Nephrology

## Abstract

Renal replacement therapy is associated with reduced physical activity. The aim of the study was to assess the relationship between regular physical activity performed with the use of virtual reality and the occurrence of symptoms of anxiety and depression in hemodialysis patients. The study involved 85 patients from the dialysis station at the Department of Nephrology, Transplantology and Internal Medicine PUM. The examined patients were randomly divided into study group and control group. The study group consisted of patients undergoing renal replacement therapy by hemodialysis, whose task was to perform VR exercises using the prototype of the NefroVR system for 20 min during hemodialysis. The control group consisted of patients undergoing renal replacement therapy by hemodialysis who were not assigned an intervention. An intragroup analysis was performed for the Beck and GAD scales. After the end of the exercise cycle in the study group there was a decrease in the score while in the control group there was an increase in comparison to the first result. The research showed that after a 3-month exercises on a bicycle with the use of low-intensity virtual reality, a decrease in depression symptoms measured by the Beck Depression Inventory was observed. The research showed that regular physical activity using virtual reality may be associated with a reduction in the occurrence of anxiety and depression symptoms in patients included in the chronic hemodialysis program.

## Introduction

Anxiety is one of the common psychiatric symptoms in patients with End-Stage Renal Disease (ESRD) treated with hemodialysis (HD). Apart from anxiety, depressive disorders also often appear. Symptoms of anxiety and depression occur in 50% of patients receiving renal replacement therapy using hemodialysis, and the frequency of their occurrence is higher than in other chronic diseases. In addition, 12–52% of patients also experience anxiety during the dialysis itself. Both the symptoms of anxiety and depression significantly deteriorate the quality of life in these patients, contribute to the occurrence of problems in social, family and work life, also increase the mortality rate^1–3^.

Virtual Reality (VR) is a term used to describe interactive computer-generated simulations that resemble real objects and events. The effect of the VR system as a supplement to rehabilitation is based on user interaction with simulated environments and receiving real-time feedback. Thanks to this, the performance of repeated functional actions is ensured, which facilitates the learning of motor functions. The use of VR gives the opportunity to change the environment and cut off the user from sounds and images that negatively affect the person's mood. Virtual Reality programs are widely used in the treatment of balance and coordination disorders, improvement of mobility and gait in the course of neurological diseases. Few studies on rehabilitation in a group of people with chronic kidney disease have been found in the literature^4–6^.

Chronic kidney disease (CKD) and long-term dialysis make these patients anxious about being less in control of their own health. Emerging serious health problems contribute to the development of negative emotions in the form of anger, dissatisfaction, and disappointment. Moreover, the patients have a problem with accepting the situation, and the anxiety about their well-being after each dialysis makes them feel helpless^7^. The initiation of dialysis significantly reduces the ability to fulfill social roles not only in the family but also in society. Therefore, financial problems and the awareness of dependence on others occur, leading to the loss of meaning in life^8^.

Chronic kidney disease and the frequency of dialysis reduce and limit physical activity^9^. Deteriorating health condition, malaise and hemodialysis treatment make it difficult for patients to undertake and perform physical activity. Moreover, the group of patients treated with renal replacement therapy usually has a negative attitude towards undertaking physical effort. The key is to encourage hemodialysis patients to exercise therapy and lifestyle modification by increasing the attractiveness of exercise, monitoring physical activity, and highlighting the benefits of regular exercise^10–13^. Regular exercise also promotes the secretion of endorphins, which may reduce depression and anxiety symptoms^2,14^.

The aim of the study was to assess the relationship between regular physical exercises performed with the use of virtual reality and the occurrence of symptoms of anxiety and depression in the group of patients treated with renal replacement therapy by hemodialysis.

## Materials and methods

**Figure 1:**
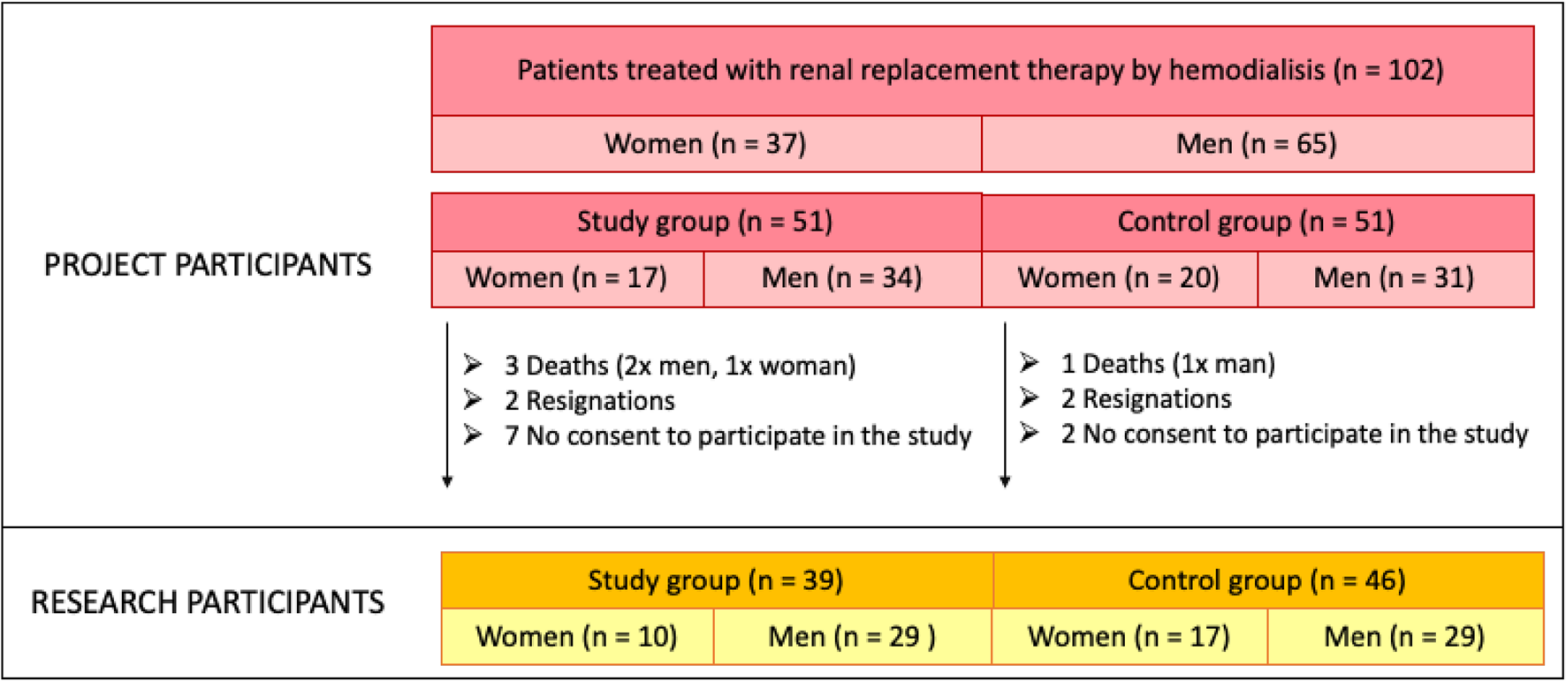
Qualification scheme for the research.

The research was performed from March 2021 to February 2022. 102 people with the fifth stage of chronic kidney disease, treated with renal replacement therapy by hemodialysis, were qualified for the study by a nephrologist. (Fig. 1). In the analysis of the surveys, it was observed that in EXAMINATION No. I, 5 patients did not complete the Beck questionnaire completely, 6 patients did not complete the GAD-7 questionnaire completely. In the EXAMINATION No. II , 6 patients did not complete the Beck questionnaire and 8 patients did not complete the GAD-7 questionnaire.

Ultimately, 85 patients treated with renal replacement therapy by hemodialysis (58 men, 27 women) participated in the study. In the study was recruited all patients were treated with renal replacement therapy by hemodialysis at the Clinic. The study group consisted of 39 people (29 men and 10 women), and the control group—46 people (29 men, 17 women).

The study and control groups included End-Stage Renal Disease patients from Department of Nephrology, Transplantology and Internal Diseases, Pomeranian Medical University, undergoing renal replacement therapy by hemodialysis due to complete absence of diuresis. Patients in both groups received 3 hemodialysis sessions per week. The average duration of one hemodialysis session was 223,85 ± 20,47 min (X ± SD) in study group and 216,52 ± 28,92 min (X ± SD) in control group.

The study group was selected based on the qualifying and disqualifying criteria for the research. The qualifying criteria for the research project were: written consent to participate in the study, complete absence of diuresis, inclusion in the program of renal replacement therapy by hemodialysis for at least 3 months (frequency three times a week), age over 18 years. The disqualifying criteria for participation in the research project were: lack of consent to participate in the study, diseases of the musculoskeletal system preventing the participation in the study, severe cardiovascular diseases (heart failure in NYHA class III or IV), acute coronary syndrome in the last three months, uncontrolled arterial hypertension, visual disturbances that cannot be corrected, poorly controlled diabetes (HbA1c levels of over 8% for 3 months), senile dementia, other neurological or mental diseases that prevent giving consent to the study or understanding the nature of the study and the conditions of participation in it; malignant tumors; surgical procedures performed in the last month; amputation of a lower limb, as a mutilation that technically precludes the possibility of conducting the examination; epilepsy.

### Qualification scheme for the research

The examined patients were randomly divided into two groups: study group and control group depending on the assigned intervention. The study group consisted of patients undergoing renal replacement therapy by hemodialysis, whose task was to perform VR exercises using the prototype of the NefroVR system for 20 min during hemodialysis. The exercises was conducted three times a week during the first (1–2) hours of HD treatment or until UF 2.5 was achieved.

The control group consisted of patients undergoing renal replacement therapy by hemodialysis who were not assigned a task.

A prototype of the NefroVR system was used for the study. The system consisted of the following components placed on a mobile platform with ballast:

- a base unit (responsible for connecting all components and running dedicated software)
- a rehabilitation rotor with a flywheel and a manually adjustable load (for exercises during dialysis)
- a panoramic screen for the patient
- a touch control screen (for the person operating the device—a doctor, nurse, or physiotherapist)
- a control kit (for the patient)—digital joystick and one button.

The device operated on the basis of audio-visual stimulation that provoked the patient's physical activity. The patient connected to the device participated in a virtual experience—in a game where movement was only possible with the rehabilitation rotor included in the device. The speed of the rotor rotation had an impact on the patient's pace in the game. The doctor/physiotherapist treating the patient was responsible for selecting the appropriate ratio of the number of rotor revolutions to speed in the game and for the physical resistance of the flywheel in the rotor. These parameters had to be matched to the patient's current health condition, so that the load was not too intense (e.g., too high heart rate or blood pressure drop/increase below/above the permitted values). In the clinical trial phase, patients had a choice of 5 mini-games, the gameplay of which was scheduled for about 15–20 min. In addition to the rotor control, the patient had additional interaction options via a digital joystick and one button. The level of interactivity available to the patient at the stage of clinical trials was limited to such an extent as to minimize the time needed to introduce the patient to the NefroVR system.

Data were collected twice: at the time of joining the research project (E0), after the third month (E3). The approval of the Bioethics Committee No. KB-0012/144/2020 of 5 October 2020 was obtained to conduct the research project.

### Description of the research performed in the study and control group

#### Survey research

At the time of entering the research, patients were asked to fill in a questionnaire prepared specifically for the study, containing demographic questions regarding the health condition and lifestyle of the participant.

The respondents were asked to complete the following questionnaires: Beck Depression Inventory, General Anxiety Disorder-7 (GAD-7) before entering the study (examination No. I) and after its completion (examination No. II).

Beck Depression Inventory (BDI) is an inventory used in the diagnosis of depression. It consists of 21 questions to which the patient answers individually. There are four variants of the answer. The level of depression is calculated from the number of points obtained after adding them up. Patients with a result of 0–10 points were classified as patients with no depression or with low mood, 11–27—with moderate depression, 28 and more—with severe depression^15–19^. The General Anxiety Disorder (GAD-7) questionnaire is a test used in the diagnosis of anxiety disorders. The test consists of seven questions relating to the behavior and well-being over the period of last two weeks on a 4-point scale. Depending on the frequency of symptoms, patients receive 0 points (not at all), 1 point (several days), 2 points (more than half the days) and 3 points (nearly every day). A total score is calculated by adding up the points for each question. The 5-, 10- and 15-point cut-offs represent mild, moderate, and severe anxiety levels^20–23^. Before performing physical activity, after 10 min of its duration and after the end of exercise, the patient was asked to indicate the level of exertion in a subjective, 20-degree BORG rating scale, where 6, 7 meant no or minimal exertion, 8, 9—very light exertion, 10, 11—light exertion; 12, 13—somewhat hard exertion; 14, 15—hard exertion, 16, 17—very hard exertion, 18, 19, 20—maximal exertion. Patients reached exertion of no more than 13 on the Borg scale (median: 8).

During each activity with the use of the NefroVR system, the doctor was responsible for monitoring the blood pressure in the study group. A value equal to or higher than 160/90 mmHg was adopted as the criterion for stopping physical effort.

### Statistical analysis

Statistical analysis was performed using the licensed program Statistica 13.0 (StatSoft, Inc. Tulsa, OK, USA). In order to characterize the group, descriptive statistics, means, standard deviations, medians, as well as multiplicities and percentages were used. The Shapiro–Wilk test was used to assess the normality of the distribution of the examined variables. The evaluation of quantitative data was performed using the Mann–Whitney U test. For the analysis of qualitative data, the X^2^ test was used, if the numerical force in a subgroup was low, the Yates correction was used. The Wilcoxon signed-rank test for dependent variables was used to evaluate the differences between the values of the scale scores before the exercise cycle and after the exercise cycle. The level of significance was p ≤ 0.01.

### Institutional review board statement

The study was conducted in accordance with the Declaration of Helsinki, and approved by the Ethics Committee of the Pomeranian Medical University (No. KB-0012/144/2020 of 5 October 2020).

### Informed consent statement

Informed consent was obtained from all subjects involved in the study.

## Results

Table 1 shows the characteristics of the group. There were no statistically significant differences between the study group and the control group.

**Table 1:** Characteristics of the group.

	Study group (n = 39)		Control group (n = 46)		p-value
Age, mean ± SD; Me	57.56 ± 17.61; 63.0		62.63 ± 15.47; 64.0		0.266*
Gender, n (%)					
Man	29	74.36%	29	63.04%	0.264
Woman	10	25.64%	17	36.96%	
Professional activity, n (%)					
No	26	72.22%	40	88.89%	0.103^^^
Yes	10	27.78%	5	11.11%	
Professional activity before starting hemodialysis, n (%)					
No	10	27.78%	21	46.67%	0.082
Yes	26	72.22%	24	53.33%	
Nature of work, n (%)					
Physical	18	47.37%	18	54.55%	0.829
Intellectual	11	28.95%	7	21.21%	
Does not work	9	23.68%	8	24.24%	
Currently smoking cigarettes, n (%)					
No	30	76.92%	35	76.09%	0.868
Yes	9	23.08%	11	23.91%	
Daily number of cigarettes, mean ± SD; Me	14.44 ± 6.13; 15.0		14.09 ± 7.41; 10.0		0.676*
Number of years without smoking, mean ± SD; Me	9.71 ± 10.95; 5.0		16.67 ± 16.17; 13.0		0.520*
Duration of single dialysis (min), mean ± SD; Me	223.85 ± 20.47; 240.0		216.52 ± 28.92; 210.0		0.110*
Comorbidities					
Diabetes, n (%)	5	14.71%	13	28.89%	0.224^^^
Arterial hypertension, n (%)	25	73.53%	32	71.11%	0.812
Ophthalmological, n (%)	8	24%	15	33%	0.484
Neurological, n (%)	2	6%	3	6.52%	0.745^^^
Treatment with renal replacement therapy by hemodialysis, n (%)	7	20.59%	8	17.78%	0.979

*n* number of patients, *SD* standard deviation, *Me* median, *p* significance level.

For data was perfomed X^2^, *test U Mann–Whitney was performed, ^^^test X^2^ with Yates correction was used.

**Figure 2:**
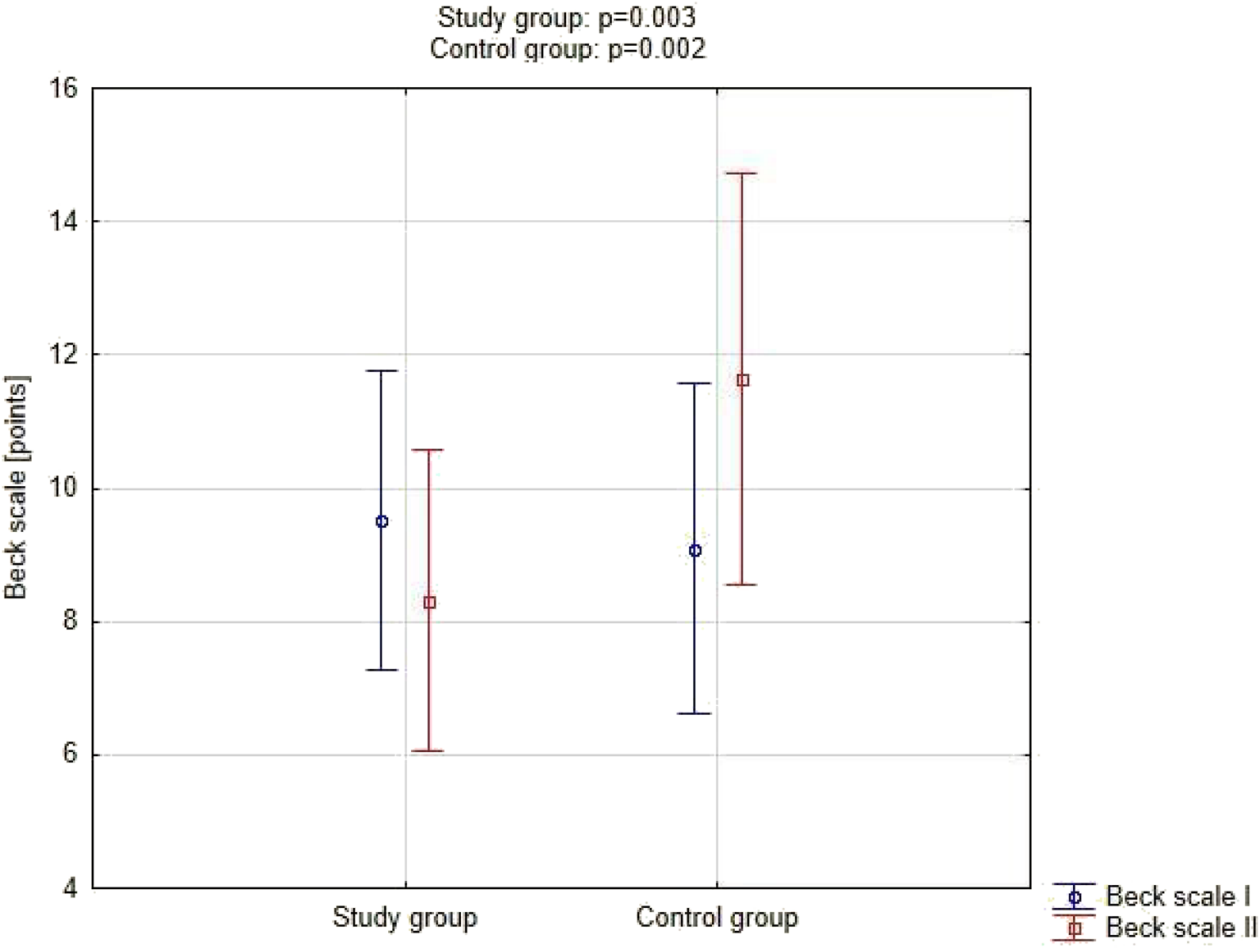
Beck scale in the study and control groups in the examination before exercise and after completion of exercise.

### Analysis between the study group and control group in the results obtained in the scales and questionnaires used in the study

The analysis of selected grouped scales in the study was performed in the study group and control group before the beginning of the exercise cycle (examination No. I) and after the end of the exercise cycle (examination No. II). In the evaluation of the scales in examinations I and II, no statistically significant differences between the groups were found. The analysis of the difference between the first examination and the second examination showed a significant decrease in scores in the study group on the Beck scale (p < 0.001) and the GAD scale (p < 0.001). The results are presented in Table 2.

**Table 2:** Assessment of the scoring of selected scales in the study group and control group in the examination performed before the beginning of the exercise cycle and after the end of the exercise cycle.

	Study group (n = 39)		Control group (n = 46)		p-value
Examination No. I					
No depression, n (%)	22	64.71%	32	71.11%	0.544
Above the norm, n (%)	12	35.29%	13	28.89%	
Mild depression, n (%)	9	26.47%	7	15.56%	0.659
Beck					
Moderate depression, n (%)	1	2.94%	2	4.44%	
Severe depression, n (%)	2	5.88%	4	8.89%	
Mean ± SD; Me	9.53 ± 6.43; 8.0		9.09 ± 8.25; 7.0		0.349
No anxiety, n (%)	16	48.48%	13	44.83%	0.773
Above the norm, n (%)	17	51.52%	16	55.17%	
GAD 7					
Mild anxiety, n (%)	6	18.18%	7	24.14%	0.954
Moderate anxiety, n (%)	5	15.15%	4	13.79%	
Severe anxiety, n (%)	6	18.18%	5	17.24%	
Mean ± SD; Me	7.15 ± 6.97; 7.0		7.10 ± 7.34; 5.0		0.642
Examination No. II					
No depression, n (%)	25	78.13%	25	56.82%	0.063^^^
Above the norm, n (%)	7	21.88%	19	42.22%	
Mild depression, n (%)	4	12.50%	11	24.44%	0.146
Beck					
Moderate depression, n (%)	3	9.38%	4	8.89%	
Severe depression, n (%)	0	0.00%	4	8.89%	
Mean ± SD; Me	8.31 ± 6.29; 7.0		11.64 ± 10.25; 10.0		0.202
No anxiety, n (%)	16	51.61%	10	34.48%	0.181
Above the norm, n (%)	15	48.39%	19	65.52%	
GAD 7					
Mild anxiety, n (%)	9	29.03%	7	24.14%	0.263
Moderate anxiety, n (%)	3	9.68%	4	13.79%	
Severe anxiety, n (%)	3	9.68%	8	27.59%	
Mean ± SD; Me	5.42 ± 5.61; 4.0		8.59 ± 7.15; 8.0		0.053
Differences between the examinations					
Beck II-I, mean ± SD; Me	−1.19 ± 1.97; 0.0		2.56 ± 5.07; 0.0		< 0.001*
GAD7 II-I, mean ± SD; Me	−1.84 ± 2.30; −1.0		1.48 ± 1.48; 1.0		< 0.001*

*n* number of patients, *p* significance level, *SD* standard deviation, *Me* median.

For data was perfomed X^2^, *test U Mann–Whitney was performed, ^^^test X^2^ with Yates correction was used.

An intragroup analysis was performed for the Beck and GAD scales. Figure 2 presents the results for the Beck scale. In the examination No. II (after the end of the exercise cycle) in the study group there was a decrease in the score (p = 0.003), while in the control group there was an increase (p = 0.002) in comparison to the first result.

**Figure 3:**
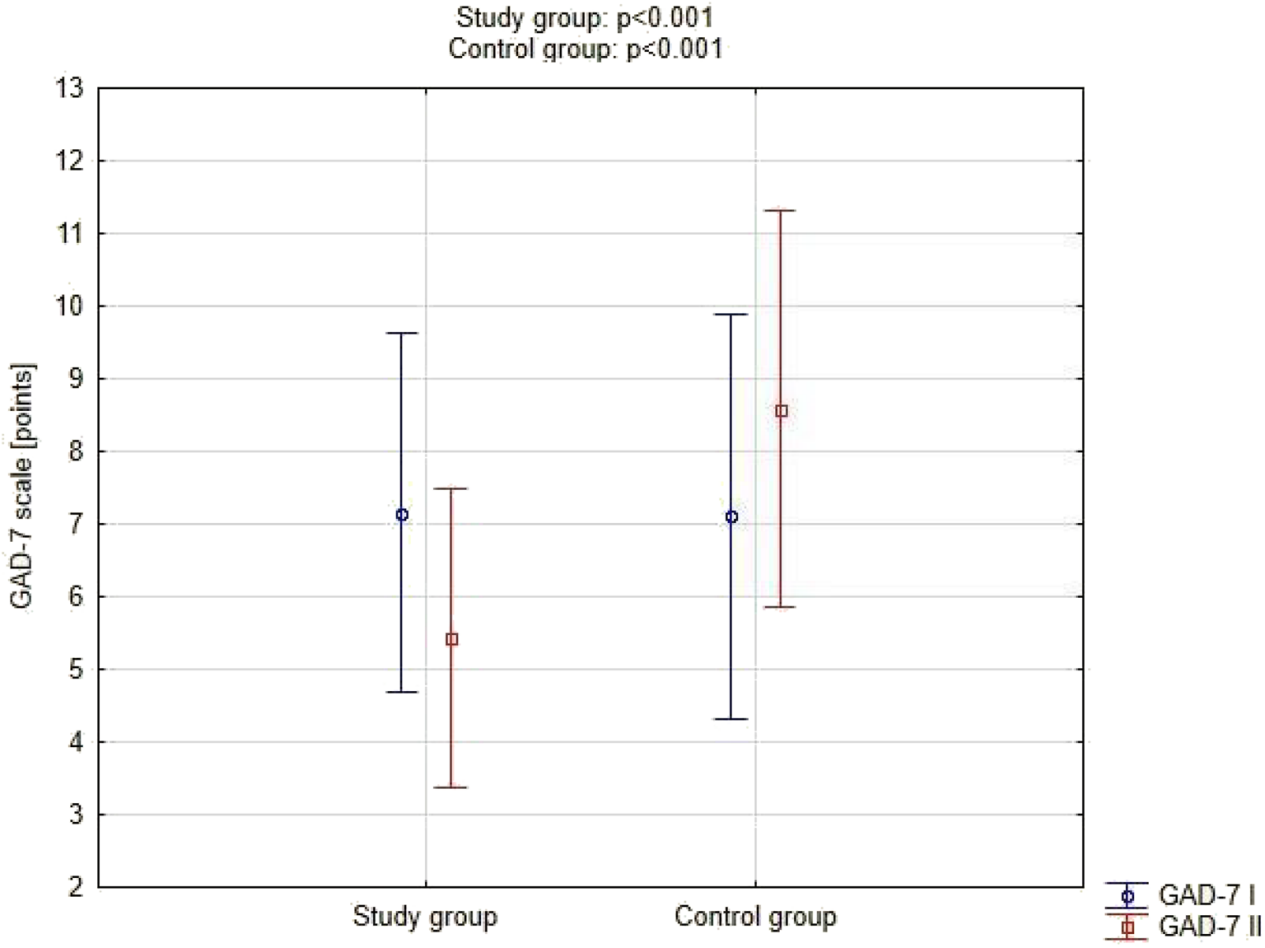
GAD scale in the study and control groups in the examination before exercise and after completion of exercise.

An intragroup analysis was performed for the GAD-7 scale. In the examination No. II (after the end of the exercise cycle) compared to the examination No. I (before the beginning of the exercise cycle), a drop in the score was observed in the study group (p < 0.001). On the other hand, there was an increase in the control group (p = 0.002). The results are shown in Fig. 3.

## Discussion

Symptoms of anxiety and depression are common in patients treated with renal replacement therapy by hemodialysis^24–26^. Factors that contribute to the emergence of depression and anxiety in this group of patients are the chronic nature of renal replacement therapy, physical symptoms (fatigue, insomnia, waiting for a possible kidney transplant) and the lack of prospects for complete recovery^26,27^.

The studies by Zhang et al. (2014) showed that depression and anxiety were more common in the group of hemodialysis patients compared to the group of healthy persons^28^. In addition, Murillo-Zamora et al. (2016) suggest that the prevalence of depression among adult patients undergoing HD is high and independent of the time since initiation of the therapy^29^. Also, Li YN et al. (2016) emphasize that symptoms of anxiety and depression are observed in the majority of hemodialysis patients^30^. The reported incidence of depression in the group of patients with End-Stage Renal Disease (ESRD) ranges from 22.8% to 39.3%^31^.

The literature describes many of the benefits of practicing and increasing regular physical activity for the health and well-being of patients with chronic kidney disease^32–35^. Despite this fact, in this group of patients there is still significantly lower level of physical activity compared to persons with normal kidney function^36,37^.

Currently, we are also witnessing a dynamic increase in the number of clinical studies that unequivocally confirm the beneficial role of physical exercises in the treatment of chronic kidney disease (CKD). However, despite the existence of documented examples of effective exercise programs considered safe and therapeutically beneficial, physiotherapy in nephrology remains limited worldwide^5,6,32,38–40^.

For several decades, scientists and clinicians have been conducting research or implementing exercise programs in hemodialysis patients, the main goal of which is to improve their health, including mental health^41^. Blumenthal et al. (2012) believe that exercises can be one of the methods of treating depression in a different group of patients^42,43^.

Own research showed that after a 3-month exercises on a bicycle with the use of low-intensity virtual reality, a decrease in depression symptoms measured by the Beck Depression Inventory (BDI) was observed. Similar results were presented in the work of Rhee et al. (2019), who assessed a six-month physical activity during hemodialysis^44^. Also, in the study of the relationship between physical activity and exercise with accessories during hemodialysis conducted by Esteve Simo et al. (2015), a reduction in the level of depression on the Beck scale was found^45^. Similarly, the meta-analysis by Hargrove et al. (2021) demonstrated a reduction in depression symptoms measured on the Beck scale due to regular physical activity in patients treated with hemodialysis^46^.

Research confirms that hemodialysis patients show symptoms of anxiety. The presented studies showed a decrease in anxiety symptoms measured by the GAD 7 questionnaire after the use of an intervention consisting in performing physical activity in virtual reality.

In the available literature, no studies were found that would be based on a similar analysis in the group of patients treated with hemodialysis. It was the first study to assess the effect of exercises with the use of virtual reality performed during hemodialysis on the occurrence of anxiety and depression symptoms in a group of HD patients. The performed preliminary examinations showed that there may be a relationship between physical activity with the use of virtual reality and the reduction of symptoms of anxiety and depression in patients treated with hemodialysis. The analysis of the literature confirmed the effectiveness of exercises with the use of virtual reality in the treatment of symptoms of depression and anxiety in other groups of patients^47–51^.

Research on the use of virtual reality in reducing anxiety symptoms has also been reviewed. Viana et al. (2020) found that playing a exercises game appears to be a useful method of reducing anxiety in healthy women^52^. In turn, Eijlers et al. (2019) showed that the use of VR effectively reduced anxiety in pediatric patients^53^. Recently, VR technology has been used in clinical settings with positive outcomes for patients experiencing severe pain and psychological phobias^54^.

During the analysis of the literature, research using virtual reality exercises, the aim of which was to improve physical fitness in groups of people treated with dialysis, has been found^5,55^. In addition, it has been shown that the use of virtual reality during intradialytic exercises increases physical activity, improves physical fitness and the quality of life of persons undergoing dialysis^4^.

Studies by other authors have confirmed the positive impact of virtual reality exercise on the quality of life. The use of video games during exercise training enables an interesting form of rehabilitation focused on fun and is an effective supplement to traditional therapy^56,57^. Studies suggest that in adults undergoing hemodialysis, the negative symptoms of depression are reduced by aerobic exercise^58^. Others also argue that symptoms of anxiety and depression in hemodialysis patients can be reduced with various treatments, including regular exercise^59^. Also, a combination of aerobic and anaerobic training during dialysis is effective for symptoms of depression in terms of mental health^60^.

There is no doubt that accumulating evidence documents the advantages and health benefits of positive emotions and psychological resources in the context of stress and illness-related trauma. VR technology can become a powerful tool for delivering mental health programs to clinical populations during outpatient treatment sessions^61^.

It is worth emphasizing that physical exercises with the use of virtual reality during hemodialysis treatment means: the possibility of using the time that the patient spends in the dialysis center (no additional time is required outside of dialysis), attraction for patients (reduction of the monotony of dialysis), promotion of a healthy lifestyle, safety (medical supervision of the staff and availability of medical equipment) and improvement of mental health.

### Limitations

This study has many limitations. Due to the COVID-19 pandemic, it was conducted in one dialysis center and a small number of participants was recruited.

The results obtained were also affected by the deteriorating health of CKD patients, renal replacement therapy and the presence of various comorbidities. In the study of patients with chronic kidney disease treated by hemodialysis, it was difficult to find persons willing to participate in this project to assess their physical activity. It was also necessary to properly select physical activity in a way that would allow the examined persons to perform the recommended intervention. The size of the study group during the research project was reduced for independent reasons, such as kidney transplants and deaths among project participants. In the conducted study, the psychological aspect of the introduction of virtual reality could have made hemodialysis patients more willing to perform physical activity. This could have been an independent factor affecting the course of the study. In the continuation of the research, it is necessary to increase the size of the study group, which would allow for a more accurate analysis of these relationships.

## Conclusions

The research showed that regular exercises using virtual reality may be associated with a reduction in the occurrence of anxiety and depression symptoms in patients included in the chronic hemodialysis program. It is crucial to encourage patients to perform physical activity in virtual reality due to the possibility of improving their mental health. It is worth considering performing similar studies not only in the group of hemodialysis patients, but also among patients treated with other methods of renal replacement therapy.

## Data Availability

The pooled data that support the findings of this study are available from the first author, A.T-S., upon reasonable request.
